# Adenoid Hypertrophy Risk in Children Carriers of G-1082A Polymorphism of *IL-10* Infected with Human Herpes Virus (HHV6, EBV, CMV)

**DOI:** 10.3390/life12020266

**Published:** 2022-02-10

**Authors:** Iuliia Lomaeva, Anna Aghajanyan, Liudmila Dzhaparidze, Olga Borisovna Gigani, Leila V. Tskhovrebova, Olga Olegovna Gigani, Valentin I. Popadyuk

**Affiliations:** 1 Department of Otolaryngology, Institute of Medicine, Peoples’ Friendship University of Russia (RUDN University), 117198 Moscow, Russia; popadyuk_vi@pfur.ru; 2 Medical Clinic “Viterra”, 117279 Moscow, Russia; dzhaparidze.l@viterramed.ru; 3 Department of Biology and General Genetics, Institute of Medicine, Peoples’ Friendship University of Russia (RUDN University), 117198 Moscow, Russia; agadzhanyan_av@rudn.ru (A.A.); gigani-ob@rudn.ru (O.B.G.); tskhoverbova-lv@rudn.ru (L.V.T.); gigani-oo@rudn.ru (O.O.G.); 4 Federal Research and Clinical Center of Physical-Chemical Medicine of Federal Medical Biological Agency, 119435 Moscow, Russia

**Keywords:** human herpesviruses 6 (HHV6), human herpesvirus 5 (CMV), human herpesvirus 4 (EBV), rs1800896 *IL-10*, adenoid hypertrophy, children

## Abstract

Adenoid hypertrophy (AH) is considered one of the most common diseases in the ear, nose and throat (ENT) practice. The cause of adenoid hypertrophy in children is still unknown. The main aim of the current study was to investigate *IL-10* (interleukin 10) gene polymorphisms and human herpesviruses 6 (HHV6), cytomegalovirus (CMV), and Epstein–Barr virus (EBV) infections in children with AH. A total of 106 children with adenoid hypertrophy and 38 healthy children aged 2–11 years were included in this study. All children with adenoid hypertrophy were divided into three subgroups depending on the adenoid size. The viruses were determined via quantitative real-time polymerase chain reaction (PCR) using commercially available kits (QIAGEN, Germany). HHV6 was more frequently detected in patients with AH compared with CMV and EBV. Among the three subgroups of children with AH, HH6 and EBV were prevalent in the children with the largest adenoid size. The frequency of genotype GG tended to be higher in the control group of children. We found significantly higher frequencies of the G allele and GG and GA genotypes for *IL-10* rs1800896 in the subgroup of children with the smallest size of adenoid compared with other subgroups. In conclusion, HHV6 and EBV infection could contribute to the adenoid size. The genotype GG for *IL-10* rs1800896 could contribute to the resistance to adenoid hypertrophy and the spread of the adenoid tissue.

## 1. Introduction

The adenoids (nasopharyngeal tonsils) are part of Waldeyer’s ring of the lymphoid tissue. Adenoid hypertrophy is considered one of the most common diseases in ENT practice. Adenoid hypertrophy (AH) occurs physiologically in children aged between 2 and 12 years but may become problematic when tissue size becomes excessive for the pharyngeal space it occupies [1]. The adenoids tend to atrophy by the age of 16 years [2]. This disease can lead to the obstruction of the upper airway and, as a result, nasal breathing difficulties, which can be associated with sleep disorders, hyponasal speech, and snoring [1]. Due to its anatomic location, adenoid tissue is linked with different pathogenic factors. Adenoid tissue participates in the mucosal immune system of the pharynx [3].

Monocytes, B and T cells, dendritic cells, and NK cells are introduced in the adenoid tissue. As a result, the adenoid takes part in the humoral and cellular responses [4]. By secreting antibodies, B cells play an important role. At the same time, different B cell subsets are able to downregulate immune responses [5]. Cytokines have the capacity to control lymphocyte stimulation, activation, proliferation, and differentiation [6]. Prior studies showed that interferon-γ (IFNγ), interleukins (IL-2, IL-4, IL-10, and IL-12), and cachectin-α (TNFα) can control and enhance the local immune response in adenoid tissue [7]. Activating CD8+ T cells, IL-10 can exert immunostimulatory effects on B cells [8]. Some results showed that IL-10 had a direct effect on B cells, including the prevention of apoptosis, enhancement of proliferation, and differentiation of plasma cells [9]. Moreover, increasing levels of proinflammatory cytokines, including IL-10, were found in children with chronic tonsillitis and adenoid hypertrophy [10]. 

The IL-10 cytokine provides an important immune regulatory and anti-inflammatory role. The IL-10 cytokine is encoded by the interleukin 10 gene (*IL-10*), which is highly polymorphic. The *IL-10* genetic polymorphism is in the proximal promoter region, including rs1800896, rs1800871, and rs1800872, forming distinct haplotypes associated with IL-10 production [11].

The evidence suggested that adenoid tissue can be a reservoir of bacteria and viruses due to the adenoids’ specific location [3]. As a result, cytomegalovirus (CMV), herpesvirus type 6 (HHV6), and Epstein–Barr virus (EBV) were detected in children’s tonsils and adenoids [12,13]. The members of the *Herpesviridae* family are categorized into *Alphaherpesvirinae* (α), *Betaherpesvirinae* (β), and *Gammaherpesvirinae* (γ) subfamilies [14]. Herpes simplex virus 1, 2, and varicella zoster are related to the *Alphaherpesvirinae* subfamily, while CMV, human herpesviruses 6 and 7 belong to the *Betaherpesvirinae* subfamily [15]. At the same time, the *Gammaherpesvirinae* subfamily includes EBV and human herpesvirus 8. During their life, all humans become infected with one or more herpesviruses [16]. Characteristically, herpesviruses persist in the host as a lifelong infection following a primary infection. At the same time, severe disease and mortality caused by *Alphaherpesvirinae* (α) and *Betaherpesvirinae* (β) subfamily are rare [16].

So far, studies have mainly focused on *IL-10* genetic polymorphisms and their association with neoplastic diseases [17,18,19,20]. Therefore, some studies raise the possibility of *IL-10* rs1800872 and *IL-10* rs1800896 polymorphisms as genetic biomarkers of gastric cancer [17]. Another study suggests that the *IL-10*-1082 G allele could correlate with the presence of squamous cell carcinoma [18]. According to other data, *IL-10* polymorphisms may play a significant role in the development of prostate cancer [19]. A role of *IL-10* (rs1800896-1082G/A) genetic polymorphism in resistance to herpesvirus infections was reported [21]. Additionally, susceptibility to herpes zoster was suggested to be genetically determined [22]. Limited and preliminary work was done to assess the role of *IL-10*-genetic polymorphism and HHV6, CMV, and Epstein–Barr virus infection in adenoid hypertrophy in children. Therefore, the main aim of the current study was to evaluate the role of *IL-10* rs1800896 polymorphism and HHV6, CMV, and EBV infections in children with AH.

## 2. Material and Methods

### 2.1. Patients 

All participants in this study were from Moscow, Russia. A total of 144 patients of both sexes who had been examined in an otolaryngology department of Viterra Clinic with a pre-diagnosis of adenoid hypertrophy between December 2019 and April 2020 were included in this study. Of these 144 children, 80 (55.5%) were boys and 64 (45.5%) were girls. All children were between 2 and 11 years old. The average age was 6.03 ± 1.05 years. According to the results of the endoscopic nasal examination and lateral radiographs, children were divided into two groups. The main group included patients with AH (*n* = 106) and the control group included patients without AH (*n* = 38).

The main group with AH was divided into three subgroups according to the adenoid size. The size of the adenoid was classified into three categories according to the distance between the vomer and the adenoid tissue [23]. The first subgroup (*n* = 28) included patients with grade 1 AH, where adenoids occupied only the upper segment in the rhinopharyngeal cavity. The second subgroup (*n* = 39) included patients with grade 2 AH, where adenoids occupied the upper half of the rhinopharyngeal cavity. The third subgroup (*n* = 32) included patients with grade 3 AH (maximum adenoid hypertrophy), where the adenoid hypertrophy extended over the rhinopharyngeal cavity with choanal obstruction [23]. The AH diagnosis was made with physical examination findings, including nasal endoscopy or lateral cephalometric X-ray findings and a characteristic history (snoring, chronic mouth breathing, sleep apnea, and otitis media). A total of 104 patients underwent flexible nasopharyngoscopy and 40 patients underwent a lateral cephalometric X-ray exam. We excluded such patients who had an acute infection in the nose, palate, or nasopharynx. The assessment of each patient included their history and a symptom questionnaire. We received the informed consent of the parents before their children participated in this study. The study was conducted in accordance with the rules of the Declaration of Helsinki, and the protocol was approved by the Local Ethics Committee of the Institute of Medicine of the RUDN University (N1, 20 September 2018).

### 2.2. Instrumental Diagnostics

#### 2.2.1. Lateral Neck Radiographic Study

A previous study showed that using lateral X-rays of the neck, despite being a non-invasive procedure, remains a very reliable and valid diagnostic test for the evaluation of hypertrophied adenoids [24]. Furthermore, some studies showed that there was a good agreement between X-rays and endoscopic findings [25]. The standard technique was used to perform lateral neck radiographs for airway patency assessment. The radiographs were obtained with the children in the supine position and their neck slightly extended by using a digital radiography system Sitec DigiRAD-FP (Japan). The adenoidal–nasopharyngeal ratio (AN ratio) expresses the adenoid size; these ratios were obtained using simple linear measurements from lateral skull radiographs [26]. The AN ratio was calculated as the ratio of the distance between the outermost point of the anterior convexity of the adenoid shadow and the straight part of the anterior margin of the basic occiput to the distance between the sphenobasioccipital synchondrosis and the posterior end of the hard palate [27].

Out of the 40 X-ray cases, 10 patients had small adenoids (25%), 16 cases (40%) had moderate degree enlargement, and 12 cases (30%) had large adenoids on the X-ray findings. Adenoid hypertrophy was not found in two patients with a previous adenoidectomy history (5%). 

#### 2.2.2. Flexible Nasopharyngeal Endoscopy

Fiber-optic endoscopy is extremely useful in the assessment of adenoid size [28]. However, this technique might not be suitable for all patients because it requires the cooperation of the child. Fiber optic video rhinoscopy (Pentax Europe GmbH, Hamburg, Germany) was used to obtain a full choanal image by the same otorhinolaryngologist in all evaluations. Before performing nasal flexible endoscopy, topical anesthesia and vasoconstriction were used in all patients using a topical solution consisting of 5% xylocaine and 0.5% phenyl ephedrine without any sedation. The size of the adenoid was classified into three categories according to the distance between the vomer and the adenoid tissue [23]. Out of 104 endoscopy cases, adenoid hypertrophy was found in 61 cases, including 18 patients (17%) with a grade 1 adenoid size, 23 patients (22%) with a grade 2 adenoid size, and 20 patients (19%) with a grade 3 adenoid size. Adenoid hypertrophy was not found in 43 cases (42%), including 38 patients in the control group and 5 patients with a previous adenoidectomy history.

### 2.3. Questionnaire

In the questionnaire, the study subjects’ parents were asked whether their children had an allergic disease, a drug allergy, a food allergy, a chronic disease, how often their children got sick during the year, and the duration of breastfeeding.

### 2.4. Molecular Genetic Study

We investigated the polymorphism G-1082A (rs1800896) of the *IL-10* gene (location: 1q32.1).

Moreover, we investigated three viruses (Table 1).

Epstein–Barr virus (EBV) is also known as human herpesvirus 4 (HHV-4). The target cells of EBV include B cells and epithelial cells [29]. Cytomegalovirus (CMV) is also referred to as human herpesvirus 5 (HHV-5). The primary target cells of CMV are epithelial cells, monocytes, and lymphocytes [30]. Human herpesvirus 6 (HHV-6) belongs to the Roseolovirus genus of the *Betaherpesvirinae* subfamily. HHV-6 species are divided into two variants: HHV-6A and HHV-6B [31]. Both HHV-6A and HHV-6B display a tropism for CD4^+^ T lymphocytes [32].

#### 2.4.1. Extracted Total DNA

For genotyping, the genomic DNA from the peripheral blood of children was extracted using a blood genomic DNA manual extraction kit (Syntol, Moscow, Russia) in accordance with the protocol. The volume for DNA extraction was 200 µL and the DNA output was 10 µg.

Nasal swab specimens were collected for the study of viruses. Nasal secretion was collected with a sterile rod nasal swab, following the rules of asepsis.

#### 2.4.2. Polymerase Chain Reaction

To study the *IL-10* G-1082A (rs1800896) gene polymorthism, we used the real-time PCR method by utilizing commercially available kits (Syntol, Moscow, Russia). Each set uses two allele-specific probes that allow for the detection of two alleles of the polymorphism. The use of allele-specific PCR in one test tube reduced the number of reactions and the competition of allele-specific primers at annealing on the matrix, and allowed for better allele separation of the homozygous genotypes. Fluorescence growth was observed in the FAM dye channel to detect the wild-type sample “normal” genotype G/G. Fluorescence growth was observed in the FAM and R6G dye channel when detecting heterozygote genotype G/A. Fluorescence growth was observed in the R6G dye channel when detecting mutant type gomozygote genotype A/A. The conditions of the qPCR was the following: 95 °C, 3 min; 95 °C, 15 s, 60 °C, 40 s, 40 cycles. We used a thermocycler CFX96 (Bio-Rad, Hercules, CA, USA) with CFX Manager TM software.

The viruses were determined quantitative real-time PCR in accordance with the protocol (the artus HHV-6 RG PCR Kit QIAGEN, Hilden, Germany). Probes specific for HHV-6A DNA were labeled with the fluorophore FAM™, while probes specific for HHV-6B DNA were labeled with a fluorophore that displays the same characteristics as Cy5. The probe specific for the internal control was labeled with the fluorophore JOE. The CMV viral load was performed in the first positive plasma samples (multiplex PCR) using the artus CMV RG PCR kit (Qiagen, Hilden, Germany) and Rotor-Gene Q (RGQ) instruments according to the manufacturer’s recommendations. The detection of EBV DNA was performed using the artus EBV RG PCR Kit.

### 2.5. Statistic

We used the R-language and SPSS version 20 statistical software to analyze our data. Comparative analyses of the genotype and allele frequencies between the groups were carried out with the chi-square test and Fisher’s exact test. Differences were considered significant when *p* < 0.05. The odds ratio (OR) and 95% confidence interval (CI) were calculated.

## 3. Results

### 3.1. Patients

**Figure 1:**
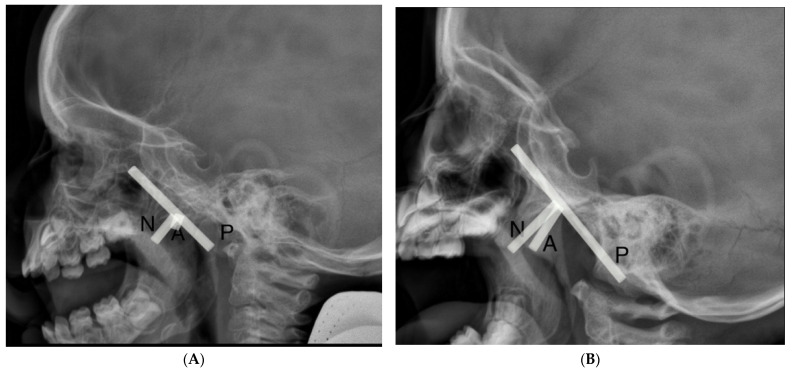
Lateral cephalometric X-ray findings (**A**) nasopharynx without adenoid hypertrophy, (**B**) nasopharynx with adenoid hypertrophy. The adenoidal–nasopharyngeal ratio was calculated as the ratio of the distance between the outermost point of the anterior convexity of the adenoid shadow (A) and the straight part of the anterior margin of the basic occiput (P) to the distance between sphenobasioccipital synchondrosis and the posterior end of the hard palate (N).

A total of 104 patients underwent flexible nasopharyngoscopy and 40 patients underwent lateral cephalometric X-ray exams. In Figure 1, we present lateral cephalometric X-ray findings, where the left side presents the nasopharynx of the patient without adenoid hypertrophy and the right side presents the nasopharynx with adenoid hypertrophy.

### 3.2. Questionnaire

**Figure 2:**
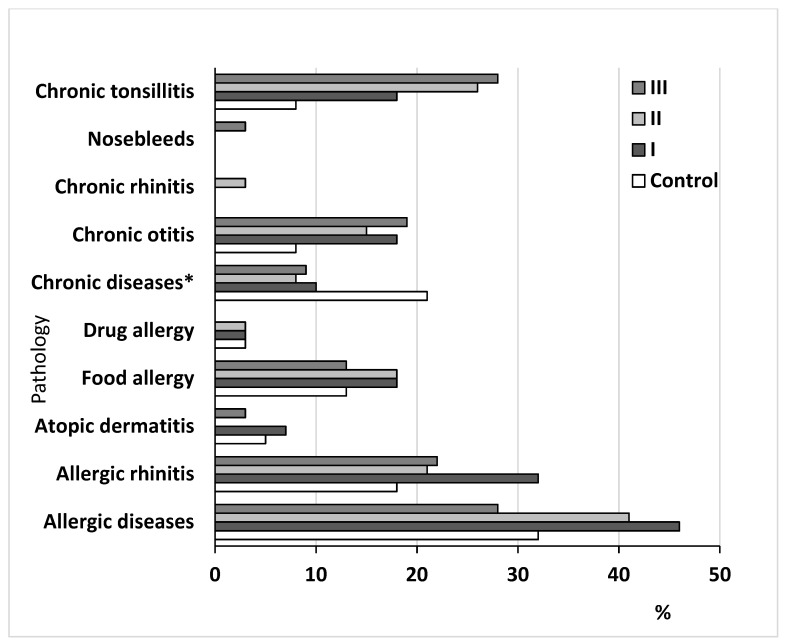
Clinical characteristics of the studied groups of children. Note: control group compared with the main group, including the I, II, and III subgroups of patients. Note: * chronic diseases excluding ENT diseases.

We found no significant differences between the groups with AH and without it for drug allergy, nosebleeds, and atopic dermatitis (Figure 2). Significant differences were found for allergic diseases, including allergic rhinitis and food allergy, chronic otitis, and chronic tonsillitis. Allergic diseases were more common in patients with adenoid hypertrophy (main group) compared with patients without adenoid hypertrophy. Allergic disease prevailed in the first and second subgroups. Among the allergic diseases, the incidence of allergic rhinitis alone was higher in children with AH (main group) than without AH (control group). The percent of allergic rhinitis was greater in the first subgroup of patients with AH compared with the second and the third subgroups.

### 3.3. Virus Infection Analysis

A total of 144 nasopharyngeal secretion samples were collected and observed to determine human herpesvirus-6, Epstein–Barr virus, and cytomegalovirus.

We detected at least one virus in 81 of the 144 patients (56%). Among the 81 patients with positive results for any herpes virus, 31 (38.3%) had over one herpes virus in patients under 11 years old. Taking into account our results of virus detection altogether, HHV6 was found most often, where it was present in 80 patients (55.6 %), followed by EBV in 22 patients (15.3%) and CMV in 21 patients (14.5%).

**Figure 3:**
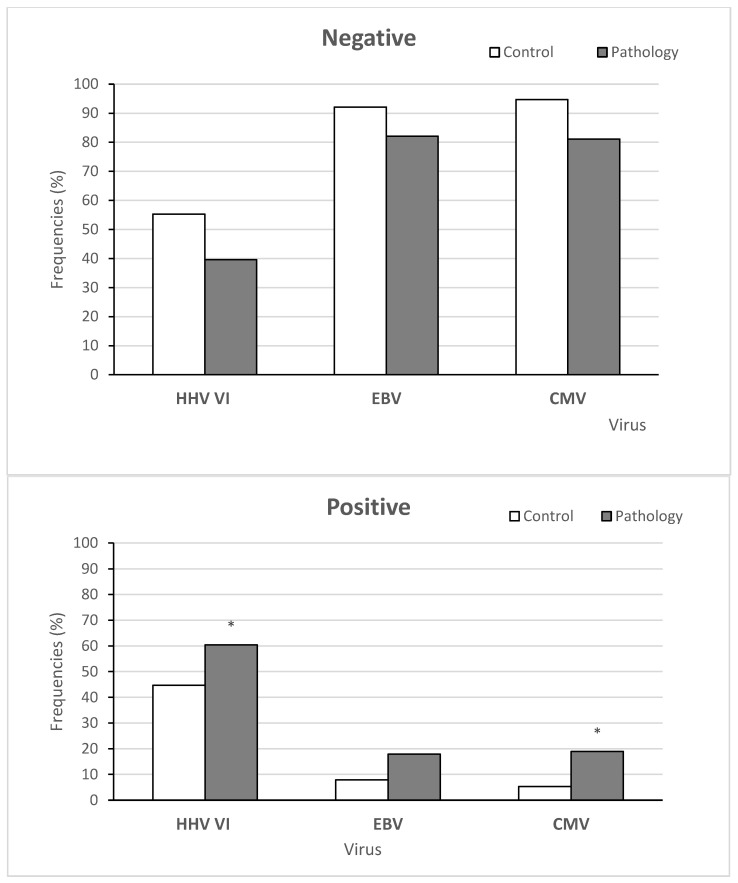
The frequencies of viral infection in the control and main groups. Note: More than one virus was detected in 31 patients in the control and main groups. Note: * significant differences between the main and control groups, i.e., *p* < 0.05.

Figure 3 shows the frequency of CMV, EBV, and HHV6 infection incidence in the control group and children suffering from AH. The distribution of the HHV6 frequency between the pathology and control groups showed significant differences (X^2^ = 3.9298, *p*-value = 0.04744, OR = 1.83, 95%CI = 1.046–3.214). Moreover, the distribution of the CMV frequency between children in the control group and main group also revealed significant differences (X^2^ = 8.0019, *p*-value = 0.004673, OR = 4.457, 95%CI = 1.593–12.468). However, significant differences were not found for EBV (X^2^ = 3.5809, *p*-value = 0.05845, OR = 2.524, 95%CI = 1.042–6.113). 

**Figure 4:**
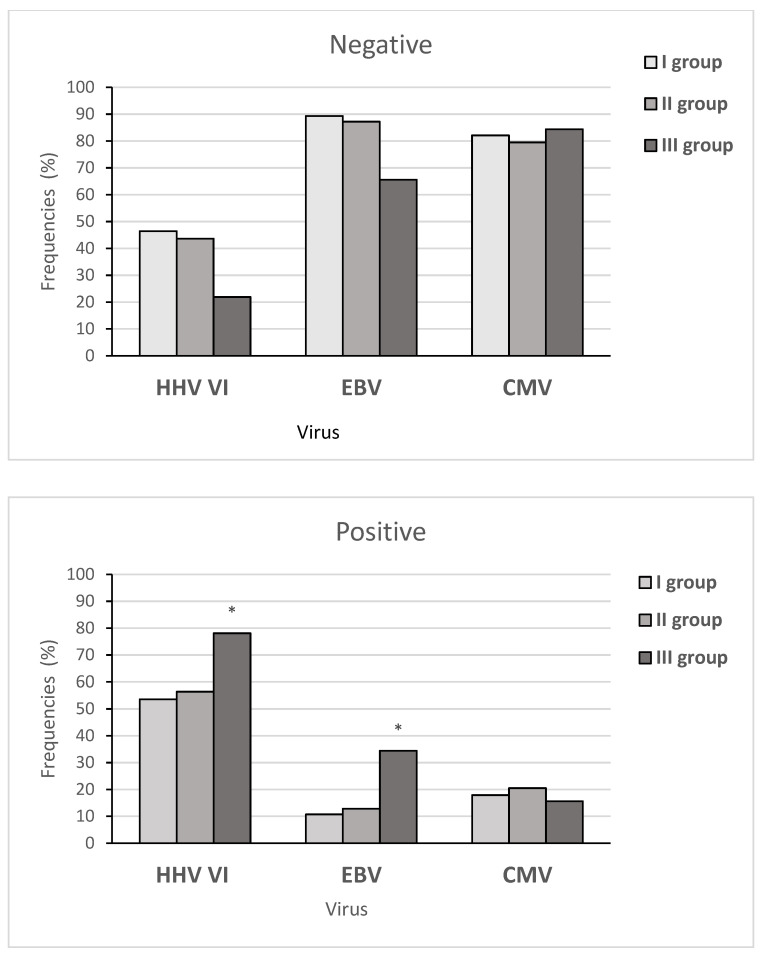
The frequency of viral infection in the three subgroups of children in the main group. Note: * significant differences between the three subgroups. Note: more than one virus was detected in 27 patients with AH.

Figure 4 shows the frequency of virus-negative and virus-positive results between the three subgroups of children. The HHV6 frequencies in the three subgroups of patients showed significant differences (X^2^ = 15.16, *p*-value = 0.0005107), as well as the EBV frequencies (X^2^ = 20.818, *p*-value = 0.02). At the same time, we did not find any significant differences in spreading CMV between these subgroups (X^2^ = 0.29538, *p*-value = 0.8627).

### 3.4. rs1800896 Polymorphism of IL-10 Gene

**Figure 5:**
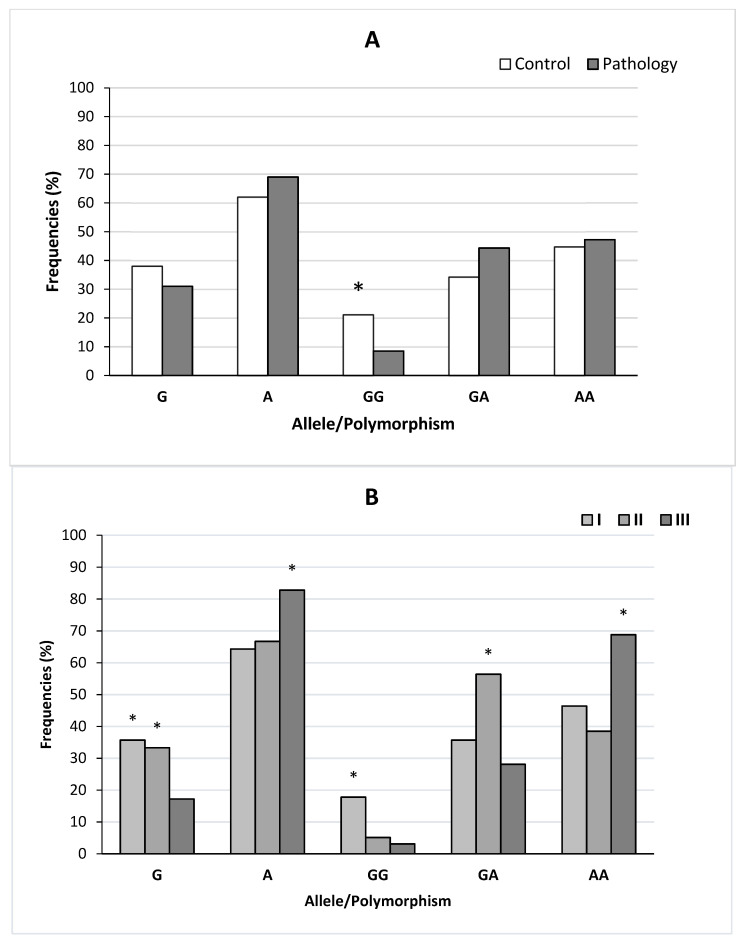
Frequencies of alleles and genotypes for rs1800896 polymorphism of the *IL-10* gene in the control and main groups (**A**) and the children of the three subgroups (**B**). Note: seven patients had a previous adenoidectomy history. Note for (A): * significant differences between the main and control groups, i.e., *p* < 0.05. Note for (**B**): * significant differences between the three subgroups of children in the main group, i.e., *p* < 0.05.

Genotype distributions for of *IL-10* rs1800896 in the control group and children with AH were significantly different (X^2^ = 6.71, *p* = 0.03). The frequency of the A allele showed no significant differences between the control group and children with AH (X^2^ = 0.79, *p* = 0.37). The frequency of genotype GG tended to increase in the control group in comparison with the main group of children. The results are summarized in Figure 5A.

The frequencies of the alleles and genotypes for rs1800896 polymorphism of *IL-10* gene in the three subgroups of children are summarized in Figure 5B. The genotypic distributions were significantly different between the three subgroups of patients with AH (*p* ≤ 0.002). The frequencies of the two alleles showed a significant difference between two subgroups: II and III (X^2^ = 6.04, *p* = 0.01) and I and III (X^2^ = 7.87, *p* = 0.005). Significantly higher frequencies of the G allele and GG genotypes of *IL-10* (rs1800896-1082G/A) were found in the first subgroup of children compared to the third subgroup. Significantly higher frequencies of GA genotypes were found in the second subgroup compared with the first and third subgroup of patients. The frequencies of the AA genotypes were higher in the third subgroup of patients compared with the first and second subgroups.

## 4. Discussion

This study found that the HHV6 and CMV frequencies in the control and main groups had significant differences. HHV6 was the most commonly detected virus in patients with AH compared with CMV and EBV. Among the three subgroups of children with AH, HH6 and EBV were more frequently detected in the third subgroup of children. At the same time, we did not find any significant differences in spreading CMV between the three subgroups. Some studies investigated the presence of herpes simplex virus (HSV), Epstein–Barr virus (EBV), and cytomegalovirus (CMV) in adenoid tissues of children with adenoid hypertrophy (AH) and chronic adenoiditis (CA) [33,34,35]. Herpesvirus type 8 (HHV8) in children’s tonsils and adenoids was detected and shown in a study [12]. At the same time, another study showed that EBV has a tropism through the rhinopharynx and children over 25 months have a greater chance of being infected by EBV [36]. Herpesviruses were found at a high rate in adenoid tissue of children with AH and CA. The authors of the article highlighted a potential relationship between the occurrence of AH and CA in patients and the presence of herpesviruses [33]. Our results also suggest a potential relationship between the presence of HHV6 and CMV and the occurrence of AH. Our findings suggested that HH6 and EBV could have an influence on the development of maximum adenoid size in children.

Another remarkable finding of this study was the distribution of genotypes for *IL-10* rs1800896 in the control and main groups. The frequency of genotype GG tended to be higher in the control group of children. Significantly higher frequencies of the G allele and GG genotypes were found in the first subgroup of children compared with the second and third subgroups. We found significantly lower frequencies of the G allele and GG and GA genotypes in the third subgroup of children compared with the second subgroup. Prior studies showed significantly higher frequencies of the A allele and AA and AG genotypes of *IL-10* (rs1800896-1082G/A) in patients resistant to HHV8 and CMV infection compared with infected ones [21]. Similar to this report, other studies found that the *IL-10*-1082 G allele was significantly higher in herpes zoster patients [37]. Interestingly, other studies showed that the polymorphism of rs1800896 in the *IL-10* gene may be related to the incidence of EBV and Epstein–Barr virus associated with hemophagocytic lymphohistiocytosis (EBV-HLH) in children, and the AA genotype and A allele of both sites may be the susceptible risk factors for EBV-HLH [38]. Furthermore, prior works showed that the MBL2 (SNP 49 C/T rs5030737) gene polymorphism [24]; the AG + GG and AG genotypes at TLR4-D299G [39]; Ugrp2 [40]; and the 2R, 2R, Il-1Ra and T, and T IL-1b genotypes [41] were associated with an increased risk of developing adenoid hypertrophy. Our results suggested that genotype GG for *IL-10* rs1800896 played a role in the resistance to adenoid hypertrophy and could prevent the expansion of the adenoid tissue to the maximum size.

Nasopharyngeal tonsils or adenoids are lymphoepithelial tissue, whose location represents the first line of defense against bacteria and viruses. In conclusion, we suggest that the presence of HHV6 and CMV infections has an influence on the developing adenoid hypertrophy and HHV6 and EBV could contribute to the adenoid size. The genotype GG for *IL-10* G-1082A might have a role in the resistance to adenoid hypertrophy and its expansion of the adenoid tissue to the maximum size. Future investigations also need to confirm our findings and explore the possible roles of other interleukin gene polymorphisms in AH. The search for predictors of adenoid hypertrophy continues. However, more studies are required to evaluate the role of herpesviruses in the pathogenesis of AH. Further study is needed to evaluate the *IL-10* rs1800896 polymorphism as a prognostic value for adenoid hypertrophy. Moreover, the pathophysiology of the disease is unclear. Identifying the factors that affect the immunological response would provide important data to explain the pathogenesis of AH.

## Figures and Tables

**Table 1:** Human herpesvirus types investigated in this study.

Human Herpesvirus Types	Equivalent	Target Cell
HHV-4	Epstein–Barr virus (EBV), Lymphocryptovirus	Epithelial cells, B cells
HHV-5	Cytomegalovirus (CMV)	Epithelial cells, monocytes
HHV-6A and -6B	Roseolovirus	T cells
